# Every great story seems to begin with a snake: a case report of large right atrial thrombus encountered during MitraClip procedure

**DOI:** 10.1186/s43044-025-00661-z

**Published:** 2025-07-01

**Authors:** Antonio Totaro, Vincenzo Ienco, Chiara Galluccio, Vincenzo Sacra, Antonio Pierro, Nicola Testa, Gianluca Testa, Cosimo Sacra

**Affiliations:** 1 Department of Medicine and Health Sciences “V. Tiberio”, University of Molise, Campobasso, Italy; 2 Interventional cardiology Unit, Responsible Research Hospital, Campobasso, Italy; 3 Interventional cardiology Unit, Responsible Research Hospital, Campobasso, Italy; 4 Department of Advanced Biomedical Sciences, University of Naples Federico II, Naples, Italy; 5 Radiology Department, S. Timoteo Hospital, Termoli, Italy; 6 Agostino Gemelli University Polyclinic, Rome, Italy

**Keywords:** MitraClip, Atrial thrombus, TEER, Carotid filter

## Abstract

**Background:**

TEER has revolutionized mitral regurgitation treatment, addressing clinical burden in aging patients. However, thrombotic complications may still occur.

**Case presentation:**

An 83-year-old man with severe mitral regurgitation underwent a MitraClip procedure. A large molding thrombus was observed during the procedure, despite a targeted ACT. To prevent cerebral embolization, two carotid filters were placed. The procedure was successfully completed, and mitral regurgitation was reduced. The patient was stable during the procedure, with no evidence of pulmonary or cerebral embolism.

**Conclusions:**

The case highlights the importance of close observation and multidisciplinary decision-making in managing acute thrombus during TEER. Further research is needed to establish the potential role of cerebral protection devices and the effect of anticoagulation procedures on thrombus formation.

**Supplementary Information:**

The online version contains supplementary material available at 10.1186/s43044-025-00661-z.

## Background

The MitraClip procedure is a ground-breaking therapeutic approach that has revolutionized the treatment of mitral regurgitation. Mitral regurgitation poses a significant clinical burden, particularly in the aging population, and the development of transcatheter interventions has brought this once exclusively surgical domain to the attention of interventional cardiologists [1].

Access to the left atrium and sufficient anticoagulation are necessary for the procedure. However, thrombotic complications can still happen even with heparin treatment and the right activated clotting time (ACT) [2].

## Case presentation

An 83-year-old man with a past medical history of atrial fibrillation (CHA2DS2VA 7, HAS BLED 3, in edoxaban 30 mg die), type 2 diabetes mellitus, and chronic heart failure (HFrEF) in ischemic cardiomyopathy (history of acute myocardial infarction in 2001, previous PCI of CX-OM in 2010), previous ICD implantation, presented with a history of worsening dyspnea associated with ankle swelling.

The results of the laboratory test showed normal coagulation parameters and platelet counts. The patient's eGFR was 48 ml per minute.

Transthoracic and transesophageal echocardiogram (TEE) showed severe mitral regurgitation with reduced left ventricular ejection fraction (EF 35%). The mitral valve presented a central prolapse of the posterior leaflet (P2) with chordal rupture and very eccentric regurgitant jet (video 1).

The patient was considered to be at very high risk for open heart surgery by the Heart Team (EuroSCORE score 8%). He was, therefore, recommended for a MitraClip procedure (Abbott Vascular, Santa Clara, CA, USA).

He underwent transcatheter edge-to-edge repair (TEER) under general anesthesia and TEE guidance. Edoxaban 30 mg, which he was taking for atrial fibrillation, was stopped the day before the procedure as recommended in 2021 EHRA practical guidelines.

For venous access, the right common femoral vein was utilized. An 8.5 F transeptal guiding introducer and BRK needle (St. Jude Medical Little Canada, Minnesota) were used for the transeptal puncture, which was carried out under TEE guidance. As soon as the septal puncture was successful, 100 UI/kg (7000 UI) of heparin were administered for anticoagulation. An initial ACT of 281 s was recorded, with monitoring during the procedure. The 0.035 × 260 cm Amplatz super stiff (Boston Scientific, Marlborough, Massachusetts) was positioned into the left upper pulmonary vein via the 8.5 sheath, and a 24 F steerable guide catheter (SGC) was inserted into the left atrium. At the same time, a giant, very mobile echogenic mass (36 mm × 8 mm), compatible with molding thrombus was observed attached to SGC at the site of transseptal puncture on the right atrial side, straddling, in minimal part, in the left atrium (7 mm) (Fig. 1a–b videos 2–3). Because of the possibility of pulmonary and systemic embolization of thrombus, SGC was not moved. To maintain ACT > 300 s, two heparin boluses (2500 UI) were administered repeatedly, after having adequately assessed the functioning of the venous access.

**Fig. 1:**
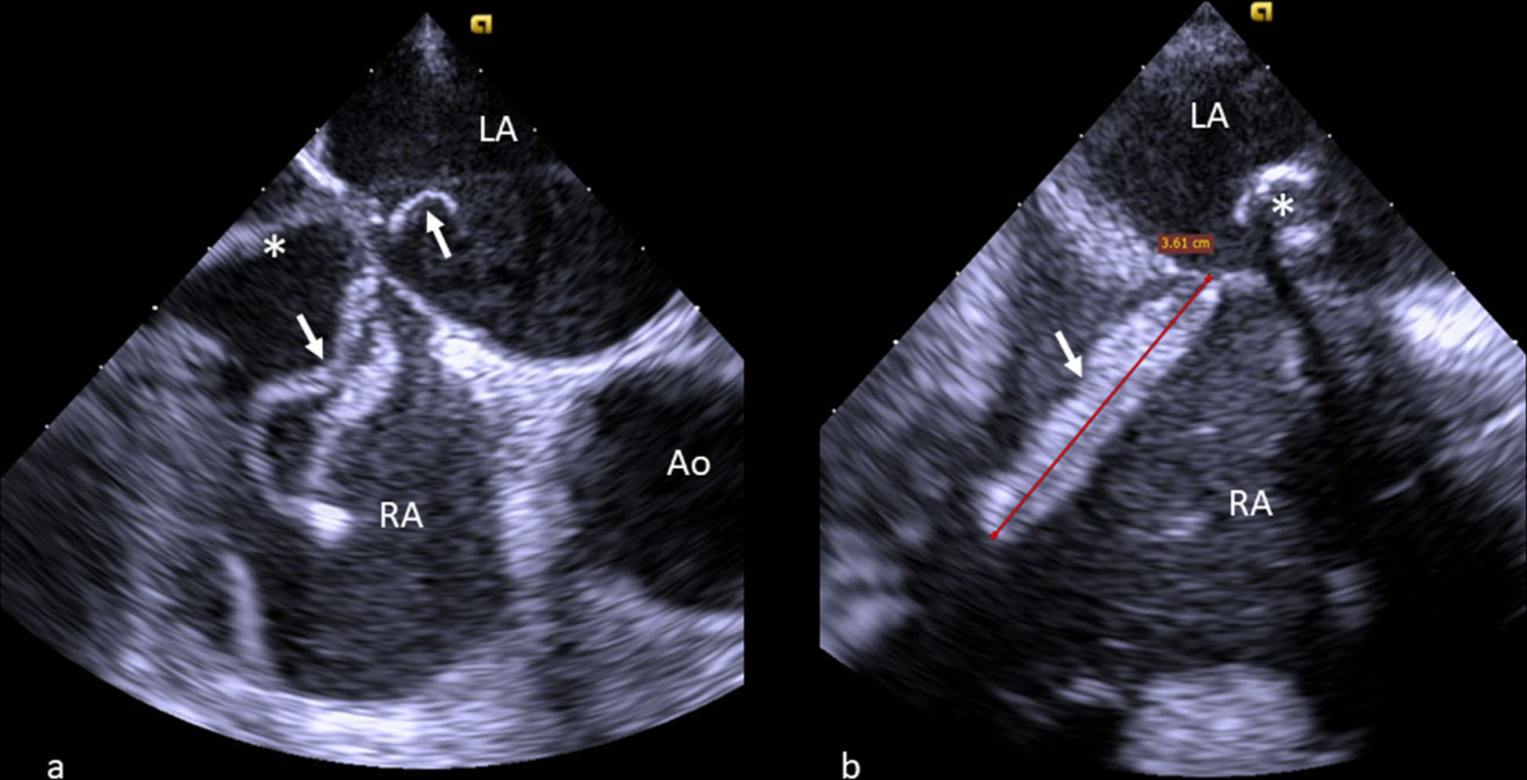
**A** Transesophageal echocardiography, ME AV SAX view. **B** Transesophageal echocardiography, ME bicaval view. LA: left atrium, RA: right atrium, Ao: aorta, * steerable guidance catheter, arrow: thrombus

In consideration of the presence of a molding thrombus, which appeared suddenly during the insertion of SGC, there was a strong suspicion that the thrombus had been dislocated by the 24 F SGC, when crossing an extensive deep abdominal vein thrombosis. For this reason, the idea of a contralateral or ipsilateral vein approach to reach the right atrium and to perform a thromboaspiration was excluded.

**Fig. 2:**
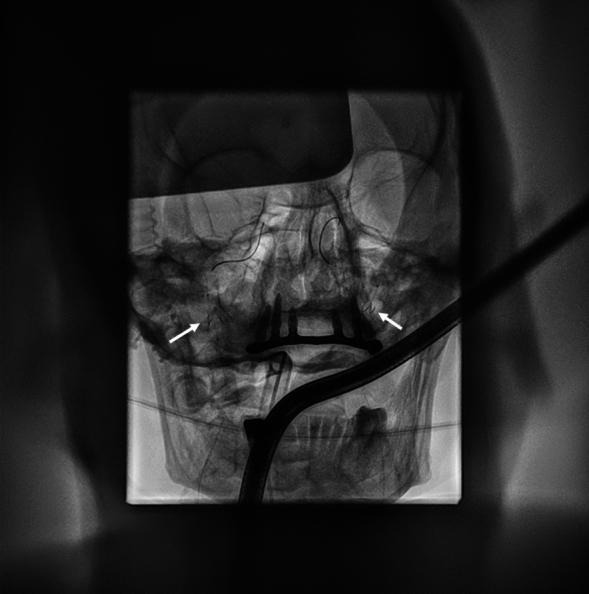
Fluoroscopy image of the deployed carotid filters inserted into the carotid arteries (arrow)

To prevent cerebral embolization, two Emboshield NAV6 Embolic Protection System (Abbott Vascular, Santa Clara, CA, USA), via bilateral femoral artery access, were placed in the carotid artery to prevent cerebral embolization (Fig.2).

When a proper anticoagulation (ACT > 350 s) was obtained, the thrombus's size was shown to be reduced (video 4).

Next, the MitraClip was successfully placed in a central position, at A2/P2 scallop of mitral valve. Mitral regurgitation was reduced from severe to mild (video 5).

At the end of procedure, thrombus partially disappeared but still partially entrapped between SGC and septum primum (video 6). In order to avoid an immediate paradoxical embolism, during SGC removal, or a future cerebral event due to the iatrogenic ASD, a Amplatzer Multifenestrated Septal Occluder 25/25 was placed while the SGC was being withdrawn (video 7).

**Fig. 3:**
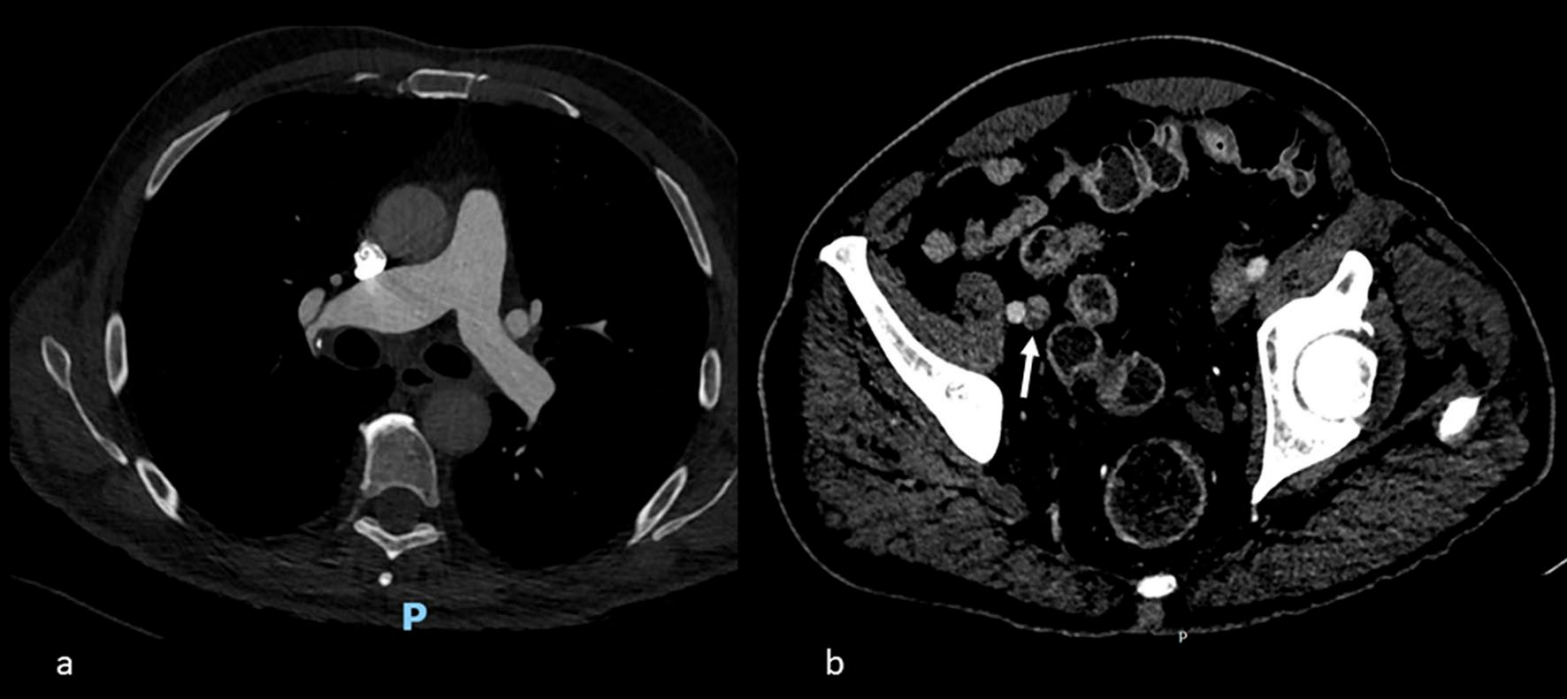
**A** CT scan: pulmonary artery. **B** CT scan: parietal thrombosis of common iliac vein (arrow)

The patient was stable during the procedure, without evidence of the right ventricular dysfunction or dilatation. He was extubated quickly. No evidence of pulmonary embolism was seen by a CT scan (Fig. 3a), while deep-vein thrombosis (common iliac veins) was found (Fig. 3b). Following the procedure, the patient was started on intravenous heparin before being switched to Coumadin. Five days later, he was discharged. The postoperative course was regular, and the patient continued his cardiological follow-up.

**Fig. 4:**
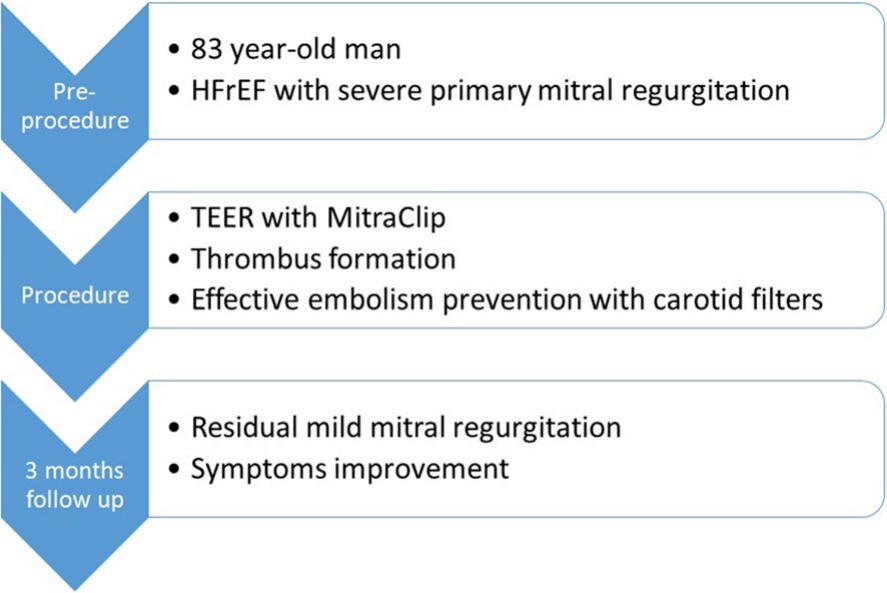
Timeline

At 3 months of follow-up, the patient was in NYHA class II, had significantly improved symptoms, and had not experienced any thromboembolic events. Residual mild mitral insufficiency was revealed by echocardiographic assessment (Fig. 4).

## Conclusions

In this paper, we describe a rare case of a huge molding right atrial thrombosis, occurring during TEER, resolved with medical therapy, without pulmonary or cerebral complications, thanks to double carotid filters and aggressive anticoagulant protocol during procedure.

TEER is being increasingly used in patients with severe symptomatic mitral regurgitation who are not surgical candidates [1]. The risk of stroke or TIA during the MitraClip implantation reaches an incidence of 2.6% [3]. Generally, the patients, in a standard protocol, received an initial bolus of heparin (100 U/Kg) immediately after positioning the transseptal sheath in the left atrium. There have been no randomized studies on the optimal time of heparin administration prior to or immediately after transseptal puncture. However, due to the possibility of embolization and ischemic stroke, periprocedural thrombus formation on the MitraClip device is an uncommon but dreaded complication.

The optimal management of acute thrombus during the MitraClip procedure is unknown. Frerker et al. suggested that the use of a cerebral protection device might be a reasonable strategy during the MitraClip implantation [4]. Huntgeburth et al. described a case of thrombus formed on SGC, despite adequate anticoagulation, which was successfully aspirated [5]. A similar solution was described by Mahmood et al., which successfully managed a right atrial thrombus using aspiration thrombectomy [6]. Another approach was described by Wolff and colleagues, which used low-dose thrombolysis to treat left atrial thrombus during MitraClip implantation [7]. Patients with a transient periprocedural thrombus had lower LVEF and tended to have worse right ventricular function and higher PASP than those without a thrombus [2].

One retrospective study of 100 patients undergoing the procedure showed that 9% of patients developed intracardiac thrombus. They were treated by flushing with heparinized saline after the transeptal sheath and needle were removed [2]. When manipulating the SGC, this technique carries the risk of systemic embolization through iatrogenic ASD.

In our case, no thrombus was found in the right atrium before the procedure. Most likely the thrombus arrived in the atrium transported by the iliac veins through the SGC. This hypothesis is supported by the shape of the thrombus (molded) and the sudden appearance in the right atrium during the entry of the SGC into it. As a result, the idea of using a vein to access the right atrium and to perform a thromboaspiration was ruled out due to the high risk of bringing additional thrombus into the atrium. This finding was confirmed by CT scan performed after the procedure. The thrombolysis option was ruled out due to the patient's high bleeding risk. It was decided not to manipulate the SGC and to wait for the resolution of the thrombus while protecting the cerebral circulation in the meantime. A dedicated brain protection system was not used due to its unavailability. The procedure was completed without complications, and, to prevent embolization of residual thrombus in the right atrium, an Amplatzer Multifenestrated Septal Occluder was positioned to treat the iatrogenic ASD.

This case report emphasizes the unusual and significant finding of a large, molded right atrial thrombus formed during transcatheter edge-to-edge repair of a severe mitral regurgitation.

Preprocedural imaging, such as duplex ultrasound of the iliac vein or IVC and ultimately CT venogram, is essential for diagnosing a venous thrombosis prior to placement of larger sheaths in high-risk patients. This is particularly crucial for procedures that need large-bore venous access, such as transcatheter mitral valve intervention. Often elderly patients, with multiple comorbidities, and sometimes with heart failure, undergoing MitraClip are at high risk for VTE. Subclinical iliac vein or inferior vena caval (IVC) deep-vein thrombosis (DVT) before placement of large sheath is highly dangerous. Comprehensive preprocedural imaging is essential for MitraClip, which requires the advancement of a large (usually 24 Fr) sheath from the common femoral vein through the iliac vein and across the interatrial septum into the left atrium. By anticipating and resolving possible venous access issues early on, it reduces the chance of serious side effects like pulmonary embolism and helps guarantee a safe and effective procedure.

In spite of the large thrombotic burden with potentially catastrophic complications, the patient did well with medical therapy only. Such a result was likely facilitated by the use of carotid double filters given preventively, thus decreasing the risk of cerebral embolization.

TEER is a successful therapeutic option for many patients, but it is associated with inherent complications, including risk for thrombus formation. Interestingly, while the optimal management of acute thrombus during TEER is still under investigation, this case teaches the importance of close observation and consideration of a multidisciplinary decision-making process.

More investigation is needed regarding the incidence, risk factors, and best practices for managing periprocedural thrombus formation during TEER. Further research is needed to establish the range of potential role of cerebral protection devices and the effect of anticoagulation procedures on thrombus formation.

## Supplementary Information

Additional file 1: Video 1 Three-dimensional color transesophageal echocardiography: surgical mitral view. Leaflet lesion and regurgitation jet

Additional file 2: Video 2 Two-dimensional transesophageal echocardiography: ME AV SAX view. Molding thrombus attached to SGC at the site of transseptal puncture on the right atrial side, straddling, in minimal part, in the left atrium

Additional file 3 Video 3 Three-dimensional transesophageal echocardiography. Molding thrombus

Additional file 4: Video 4 Two-dimensional transesophageal echocardiography: ME bicaval view. Molding thrombus

Additional file 5: Video 5 Two-dimensional color transesophageal echocardiography: Final result after MitraClip implantation

Additional file 6: Video 6 Two-dimensional transesophageal echocardiography: ME 4 chamber view. Residual thrombus at the end of procedure

Additional file 7: Video 7 Two-dimensional transesophageal echocardiography: ME bicaval view. Amplatzer Multifenestrated Septal Occluder

## Data Availability

No datasets were generated or analysed during the current study.

## Abbreviations

TEER: Transcatheter edge-to-edge repair
TEE: Transesophageal echocardiography
SGC: Steerable guide catheter
ASD: Atrial septal defect

## References

[1] Feldman T et al (2011) Percutaneous repair or surgery for mitral regurgitation. N Engl J Med 364(15):1395–1406 21463154 10.1056/NEJMoa1009355

[2] Pregowski J et al (2020) Incidence, clinical correlates, timing, and consequences of acute thrombus formation in patients undergoing the MitraClip procedure. Kardiol Pol 78(1):45–50 31719512 10.33963/KP.15056

[3] Glower DD et al (2014) Percutaneous mitral valve repair for mitral regurgitation in high-risk patients: results of the EVEREST II study. J Am Coll Cardiol 64(2):172–181 25011722 10.1016/j.jacc.2013.12.062

[4] Frerker C et al (2016) Cerebral protection during mitraclip implantation: initial experience at 2 centers. JACC Cardiovasc Interv 9(2):171–179 26723763 10.1016/j.jcin.2015.09.039

[5] Huntgeburth M et al (2014) Thrombus formation at the MitraClip system during percutaneous mitral valve repair. JACC Cardiovasc Interv 7(9):e111–e112 25129667 10.1016/j.jcin.2014.03.010

[6] Mahmood M et al (2023) Management of right atrial thrombus during mitraclip implantation: a case report and review of literature. Cardiovasc Revasc Med 47:97–99 35624011 10.1016/j.carrev.2022.05.020

[7] Wolff G et al (2019) Low-dose thrombolysis for the management of left atrial thrombus formation during percutaneous mitral valve repair. JACC Cardiovasc Interv 12(2):e9–e10 30594509 10.1016/j.jcin.2018.10.012

